# Development and Implementation of South Asia’s First Heat-Health Action Plan in Ahmedabad (Gujarat, India)

**DOI:** 10.3390/ijerph110403473

**Published:** 2014-03-25

**Authors:** Kim Knowlton, Suhas P. Kulkarni, Gulrez Shah Azhar, Dileep Mavalankar, Anjali Jaiswal, Meredith Connolly, Amruta Nori-Sarma, Ajit Rajiva, Priya Dutta, Bhaskar Deol, Lauren Sanchez, Radhika Khosla, Peter J. Webster, Violeta E. Toma, Perry Sheffield, Jeremy J. Hess, the Ahmedabad Heat and Climate Study Group

**Affiliations:** 1 Natural Resources Defense Council, New York, NY 10011, USA; E-Mails: ajaiswal@nrdc.org (A.J.); bdeol@nrdc.org (B.D.); laurenksanchez@gmail.com (L.S.); radhika.khosla@gmail.com (R.K.); mconnolly@nrdc.org (M.C.); 2 Department of Environmental Health Sciences, Mailman School of Public Health, Columbia University, New York, NY 10032, USA; 3 Ahmedabad Heat and Climate Study Group, Gandhinagar, Gujarat 380054, India; 4 Ahmedabad Municipal Corporation, Medical Officer of Health, Ahmedabad, Gujarat 380001, India; E-Mail: suhaspkulkarni@gmail.com; 5 Indian Institute of Public Health, Gandhinagar, Gujarat 380054, India; E-Mails: gsazhar@iiphg.org (G.S.A.); dmavalankar@iiphg.org (D.M.); amrutasri.nori-sarma@yale.edu (A.N.-S.); arajiva@iiphg.org (A.R.); priyadutta@iiphg.org (P.D.); 6 School of Earth and Atmospheric Sciences, Georgia Institute of Technology, Atlanta, GA 30332, USA; E-Mails: pjw@eas.gatech.edu (P.J.W.); vt25@mail.gatech.edu (V.E.T.); 7 Icahn School of Medicine at Mount Sinai, New York, NY 10029, USA; E-Mail: perry.sheffield@mssm.edu; 8 Department of Environmental Health, Rollins School of Public Health, Emory University, Atlanta, GA 30322, USA; E-Mail: jhess@emory.edu; 9 Department of Emergency Medicine, School of Medicine, Emory University, Atlanta, GA 30322, USA

**Keywords:** heat, climate change, India, vulnerability, urban, public health, adaptation, disaster preparedness, temperature forecast, extreme weather, climate events

## Abstract

Recurrent heat waves, already a concern in rapidly growing and urbanizing South Asia, will very likely worsen in a warming world. Coordinated adaptation efforts can reduce heat’s adverse health impacts, however. To address this concern in Ahmedabad (Gujarat, India), a coalition has been formed to develop an evidence-based heat preparedness plan and early warning system. This paper describes the group and initial steps in the plan’s development and implementation. Evidence accumulation included extensive literature review, analysis of local temperature and mortality data, surveys with heat-vulnerable populations, focus groups with health care professionals, and expert consultation. The findings and recommendations were encapsulated in policy briefs for key government agencies, health care professionals, outdoor workers, and slum communities, and synthesized in the heat preparedness plan. A 7-day probabilistic weather forecast was also developed and is used to trigger the plan in advance of dangerous heat waves. The pilot plan was implemented in 2013, and public outreach was done through training workshops, hoardings/billboards, pamphlets, and print advertisements. Evaluation activities and continuous improvement efforts are ongoing, along with plans to explore the program’s scalability to other Indian cities, as Ahmedabad is the first South Asian city to address heat-health threats comprehensively.

## 1. Introduction

### 1.1. Heat as a Public Health Concern in Gujarat State

The city of Ahmedabad is in the largest district in the western Indian state of Gujarat. With a population of 7.2 million and rapid growth in the real estate, automotive, and pharmaceutical sectors, Ahmedabad is among the top ten fastest growing cities in India, and is poised to become one of India’s leading metropolises by 2020 [1]. 

Ahmedabad is also one of India’s hottest cities (see Supplemental Material for additional information on its climate), and heat poses a significant public health challenge [2] when daily maximum temperatures average 45 °C (113 °F) in the pre-monsoon “summer” months of March–May [3]. Heat affects Ahmedavadis’ health through the usual pathways [4] of dehydration, acute heat illnesses such as heat exhaustion and heat stroke, and exacerbations of chronic cardiovascular and respiratory diseases. Environmental factors such as poor air quality, driven by industrial expansion that has spurred growth in cities like Ahmedabad, can worsen during extreme heat and lead to respiratory and other illnesses [5]. Heat is also known to fuel outbreaks of vector-borne diseases such as malaria, chikungunya and dengue fever, all prevalent in Ahmedabad [6], and to impact the incidence of diarrheal disease, especially among children [7].

Several factors put certain Ahmedabad populations at higher risk. Those in areas of higher population density, who have compromised safe water access, experience high ambient temperatures, and have low prevalence of protective measures such as air conditioning [8], are more vulnerable. Particularly vulnerable residents include those in the city’s migrant slums, which make up at least one-quarter of households [9]; other poor communities; the elderly and the young; and outdoor workers such as construction laborers, municipal police officers, and rickshaw drivers. In these groups, higher risk of heat-related illness appears to be due to a combination of their prolonged heat exposure, poor underlying health status, and lack of access to coping mechanisms such as air conditioning [10]. 

### 1.2. Climate Change and Extreme Heat in India

In 2010, Ahmedabad experienced its worst heat wave to date, when temperatures rose to a record 46.8 °C (116 °F), causing hundreds of heat-related deaths [11] and a significant spike in all-cause mortality. Preliminary analyses suggest that an excess all-cause mortality of at least 30% was associated with this event [12]. 

Climate change and rapid population growth are expected to amplify the problem. The effects of climate change on heat-related health outcomes are widespread globally [13]; and climate change fuels extreme heat events that may be greater in frequency, intensity and duration [14]. Mean annual temperatures across India in the last decade (between 1990–2009) have increased up to 1 °C relative to historical averages (1961–1990) [3]. Higher average annual surface temperatures and more frequent and severe heat waves are projected for South Asia as for many other regions [13,15]. 

Regardless of climate change, however, heat waves already occur regularly across South Asia, and communities that maximize current adaptations will likely deal with growing extremes more adeptly. Cities like Ahmedabad will have to create and implement adaptation strategies to avoid health impacts like those observed during the 2010 heat wave. Currently, however, there appears to be limited resources available for heat health preparedness in South Asia and very few early warning systems have been implemented. Compounding this lack of adaptation measures is a lack of information on heat stress and preparedness strategies of particular relevance to types of vulnerable populations who suffer most from the ill effects of heat exposure in Ahmedabad [16]. 

### 1.3. Origins of the Ahmedabad Heat and Climate Study Group

At the time of the 2010 heat wave, the population of Ahmedabad was no better protected than those of other major South Asian cities. Extreme heat was generally viewed as a concerning hazard but not a sustained threat to public health comparable to the city’s many other pressing health issues. The heat wave galvanized Ahmedabad’s public health community and stimulated concern about heat as a public health hazard; emerging concerns about public health adaptation to climate change provided additional motivation for pursuing adaptive action. 

The project was the next step in a developing international partnership, started in 2009, aimed at fostering public health adaptation to climate change in India. Recognizing that strong scientific research and environmental health-based governance policies are critical to protect communities from the growing threats of climate change [17], the Natural Resources Defense Council (NRDC), which has a strong presence in India, and the Public Health Foundation of India (PHFI) partnered to develop a climate health project. The partnership started as part of “The Joint Indo-U.S. Climate Change and Public Health Workshop” in Goa, India, hosted by the Indian Council on Medical Research, the U.S. Centers for Disease Control and Prevention, and the University of Michigan in August 2009 [2], along with along with expert presenters from Ramachandra University and Emory University. 

After the 2009 meeting, the team decided to pursue a heat adaptation project in Ahmedabad. The team chose the hazard of extreme heat because it has been increasingly common in the region and is expected to worsen further with continued climate change. The team chose Ahmedabad because of its dramatic 2010 heat wave and because the Ahmedabad Municipal Corporation (AMC) had strong interest, significant capacity, and a history of developing disaster preparedness in response to a deadly earthquake in 2001 (see Supplemental Material and Table S1 for information regarding city selection). The project also provided an opportunity to develop an interdisciplinary, inter-agency intervention linking preparedness and response and to expand on the limited literature on heat interventions in South Asia, potentially serving as a model for future climate change preparedness efforts. The larger context was also favorable, as the state of Gujarat has founded one of the first Climate Change Departments in India. Finally, the city was chosen because PHFI has a regional institute in Gujarat, the Indian Institute of Public Health-Gandhinagar (IIPH-G). IIPH-G and its director are proven and trusted local leaders with significant experience developing local and regional public health initiatives. By including the director and his team, the project demonstrated significant capacity to conduct scientific research and develop policy interventions. 

### 1.4. Program and Paper Objectives

The project’s overall goal was to develop a research-based heat action plan for Ahmedabad. Its specific objectives were: (1) to assess heat-health impacts of the 2010 heat wave, with a focus on vulnerable populations; (2) to use the best available evidence to develop a suite of protective measures for the city; (3) to use the 2010 heat wave to generate estimates of temperature thresholds at which management interventions should be initiated; (4) to develop a probabilistic forecasting system that could provide highly reliable temperature forecasts earlier than those that are currently available from the India Meteorological Department (IMD); and (5) to evaluate the program’s impacts and outcomes, particularly on vulnerable populations.

The goals of this paper are to provide a narrative of the project, offer details on the first heat-health action plan implemented in a South Asian city, describe some of the challenges encountered; outline anticipated next steps and highlight elements of the project that may be adapted and applied toward reducing heat-health vulnerability in other lower-resource settings. In the Methods section, we outline our approach to the project’s development and implementation and we present the project’s activities and findings related to the effort in the Results section. In the Discussion section, we review the findings in light of the published literature and implications for similar efforts in other lower-resource settings.

## 2. Methods

The project has seven overlapping but distinct phases. The first focused on *planning and conceptual model development* in which goals and objectives were clarified; this led to the development of a conceptual logic model for the activity and its implementation and evaluation. The second phase focused on *needs assessment* with key constituencies, communities, and organizations and the third on *baseline data collection.* The fourth phase focused on *coalition building and outreach* in anticipation of intervention and evaluation activities. The fifth phase focused on *intervention development* across a range of settings. The sixth phase focused on *intervention implementation*, and the seventh on *project evaluation*. These last three phases are currently ongoing. 

### 2.1. Planning and Conceptual Model Development

This phase was undertaken via structured, facilitated face-to-face meetings involving team members and important local partners, constituent representatives, and international experts. The goal was to integrate the best evidence regarding public health preparedness for extreme heat with local needs and programming preferences. The meeting series included a two-day kick-off workshop involving over 40 public health experts, scientists, policy makers, government officials, and local stakeholders from India and the United States. The findings from this meeting were summarized in a meeting report [18].

### 2.2. Needs Assessment

Needs assessment activities included characterization of population vulnerability to heat in Ahmedabad, particularly of highly-exposed or susceptible populations; evaluation of the impact of the 2010 heat wave on morbidity and mortality generally and among specific populations; assessment of the health sector’s capacity to respond to extreme heat; and characterization of the appropriate thresholds for early-warning and other interventions designed to reduce health impacts from extreme heat exposure. These assessments were done in parallel, using a variety of methodological approaches outlined in the Supplementary Material Table S2. 

This work was supplemented with other qualitative work, including a series of focus groups to develop adaptation strategies with physicians, medical social workers, government officials, and community leaders. Roundtables and semi-structured interviews were conducted with labor departments, hospital officials, media experts, and meteorological experts. The team also conducted site visits to local hospitals, ambulance and response services, the Ahmedabad Meteorological Centre, slum communities, parks and other potential “cooling centers.” Comparative research was conducted on heat mortality and morbidity research and early warning systems in the United States, Canada, China and elsewhere, as well as a comprehensive literature review. 

### 2.3. Baseline Data Collection

We collected a range of baseline data including mortality records from the registrar of births and deaths at the Ahmedabad Municipal Corporation; outpatient and inpatient attendance from city hospitals; temperature data for several decades; emergency ambulance calls for the past six years; heat vulnerability surveys with slum communities and outdoor workers; focus group discussions with government officials and health care professionals; and semi-structured interviews with key government officials. These data were analyzed using descriptive statistics; the group also conducted an in-depth analysis of the 2010 heat wave to evaluate its impacts and determine what temperature thresholds may be most useful for issuing heat warnings [12,19]. The team also undertook several field research efforts. The first was a summer of 2011 epidemiological survey of 300 households in slum communities to assess exposure and susceptibility to extreme heat [16]. A similar survey with 100 construction workers from four sites, part of an international effort to assess occupational heat exposure [20], was conducted in the summer of 2012 [21]. Associated quantitative and qualitative analysis examined the effects of the 2010 heat wave on neonates in the infant ward at the Shardaben Hospital, the main slum community hospital in Ahmedabad [22]. Results from these analyses were developed into manuscripts for peer-reviewed journals.

### 2.4. Coalition Building and Outreach

With input from local leaders and officials, the group drafted a list of key community and other contacts and initiated a systematic outreach effort. Outreach took place in person, via electronic communications and social media, and through workshops, and used products such as policy briefs, blog posts, and published research findings from the group’s early activities to engender additional interest and collaboration among key partners.

### 2.5. Intervention Development

Several interventions were developed during the project, including medical officer training and enhanced interagency cooperation. Two interventions that are central to the early warning system effort include a Heat Action Plan (HAP), and an extreme heat early warning system based on probabilistic weather forecasting. The methods used to develop these two interventions are described here.

#### 2.5.1. Heat Action Plan

The HAP was developed as an administrative tool that would define different levels of heat emergency for the city and clarify activities among the plan participants for each level. The HAP was developed to incorporate the best practices from other plans and to incorporate a robust community outreach campaign. 

#### 2.5.2. Extreme Heat Early Warning System

The HAP depends on early warnings for extreme heat. The IMD issues forecasts of extreme heat a little over a day in advance. In examining early warning systems for extreme heat developed internationally, the project team along with Ahmedabad government officials determined that developing pilot longer-term forecasts may prove useful in preparing for heat waves. The pilot forecasts were generated through an innovative hybrid dynamical-statistical temperature forecast system developed by the Georgia Institute of Technology (Georgia Tech, Atlanta, GA, USA) and the Climate Forecast Applications Network. The hybrid model uses the European Center for Medium-Range Weather Forecast (ECMWF) Variable Ensemble Prediction System (VarEPS) that is statistically post-processed and calibrated to adjust for model bias in a manner similar to previous work conducted at Georgia Tech [23,24]. 

### 2.6. Intervention Implementation

After the interventions were developed, they were first implemented in a pilot fashion in 2013. Several preparatory activities were undertaken, including circulation of the draft HAP and solicitation of feedback from government officials, medical officers, and community groups. The group also conducted a “table-top exercise” to implement the HAP with government officials during different simulated heat emergencies. Based on findings from this exercise, modifications to agency actions were made, particularly focused on interagency coordination, and an interagency communication plan was developed. Implementation success was assessed at the end of the pilot period.

### 2.7. Project Evaluation

Program evaluation includes assessment of its effects on two main target populations: organizations involved in the public health response to extreme heat, and the general population, particularly people most vulnerable to extreme heat. Formal post-implementation impact assessments on these two groups will take place in late 2014 after the first full year of formal warnings and programming. 

## 3. Results

Below we present the results by project phase. While this initiative has made efforts to be data driven, the actual numerical data is presented in detail in associated manuscripts cited below. The phases presented here, by contrast, emphasize more the intervening steps that occurred, the collaborative network that developed, and the academic, institutional, and governmental presentations and publications and institutional reports that resulted.

### 3.1. Planning and Conceptual Model Development

The group along with Ahmedabad government officials determined that the project’s overarching goals were: (a) to develop a study group of policy experts and scientists across several sectors and government agencies; (b) to assess and manage risks related to extreme heat in Ahmedabad; and (c) to facilitate long-term climate change adaptation. 

The logic model assumed that extreme heat currently poses a significant public health risk, that this risk is under-recognized and inadequately managed, and that outlining management strategies would improve public health and facilitate climate change adaptation in the long run. It also assumed that certain populations are at greater risk because of high exposure (e.g., outdoor laborers) or low coping capacity (e.g., slum dwellers) and that strategies for conveying information regarding risk (e.g., temperature forecasts, extreme heat warnings) and management strategies (e.g., health education, potable water provision, cooling centers) would reduce the risks related to extreme heat. Finally, the logic model assumed that lessons learned in the heat management project could be applied to management of other climate sensitive public health concerns.

Much of the planning took place at the 2011 kick-off workshop. The workshop proceedings were published in the report *Climate Change and Health Preparedness in India: Protecting Local Communities in Ahmedabad, Gujarat from Extreme Heat* [18] (See Supplemental Material and Figure S1 and Table S3 for additional information).

**Figure 1:**
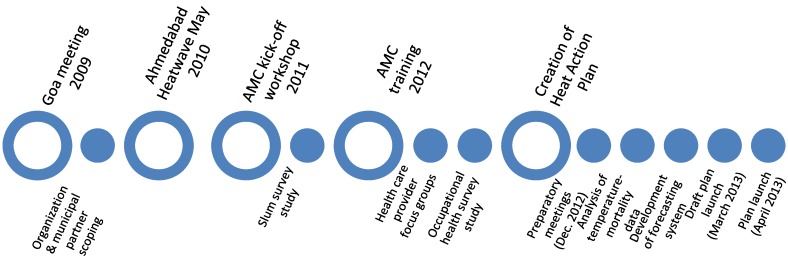
Timeline of extreme heat adaptation project components from 2009 through 2013. Rings represent key events such as kick-off meetings and the 2010 heat wave. Solid circles represent supporting activities such as partner scoping, survey studies, forecasting system development, and activities behind the creation of Ahmedabad’s Heat Action Plan.

The planning meetings determined the timeline for the remainder of the project, depicted in Figure 1.

### 3.2. Needs Assessment

The data from the needs assessment activities helped in confirming findings from the theoretical conceptual model. From this research, we prepared a series of four policy briefs for providers , government officials, community leaders and members, and workers exposed to extreme heat [25]. These documents drove priorities for baseline data collection and intervention development and, ultimately, the 2013 Heat Action Plan. The information from these activities, in turn, focused the HAP on specific, actionable, and high-priority action items. Additionally, the needs assessment helped the group clarify its highest research priorities and identify burdensome health concerns that have not been adequately studied in the area. 

The needs assessments led to multiple important conclusions:

- extreme heat is an under-appreciated public health hazard in Ahmedabad (and probably in other similar cities); excess mortality associated with the 2010 heat wave of at least 30% occurred [12,19], which was on par with similar events elsewhere [26];
- significant parts of the public health and health care delivery sectors are not adequately prepared for extreme heat events but the 64 Urban Health Centers, 26 hospitals, and over 1,000 link workers that make up the AMC health care system represent substantial human resource potential in the activation of an HAP [27];
- life-time prevalence of heat illness is greater than 30% among slum dwellers, and protective measures such as air-conditioning are rare in slum communities [28]; risk for mortality and other health impacts appears to increase significantly around 42 °C;
- outdoor workers are a particularly vulnerable group when considering extreme heat where an estimated 10 percent are hospitalized at least once during the summer for heat-related issues and 50 percent wear thick cotton clothing for work, which generally increases the effects of heat stress [29];
- neonates are another vulnerable group given their immature physiology and large body surface to mass ratio but that simple interventions as implemented by one local hospital can be protective [22]; and
- established government definitions of heat waves and forecasting lead times are not well suited for public health activities related to extreme heat [30].

Four policy briefs cited above [27,28,29,30], summarizing some of the initial findings, were developed by the group and focused on recommendations for healthcare providers, slum inhabitants, outdoor workers, and government officials.

### 3.3. Baseline Data Collection

The group was successful in obtaining access to relevant baseline data on daily minimum and maximum temperatures, mortality, hospital admissions, daily ambulance calls, and historical extreme weather events in India. This data was analyzed and, as appropriate, analyses were prepared as conference presentations and journal manuscripts [12,19]. Findings corroborated the anecdotal evidence of significant health impacts from the extreme heat demonstrating at least a 30% increase in mortality during the 2010 heat wave *versus* comparable time periods during non-heat wave events. This process of assembling and analyzing data has consolidated the group’s interest in taking a data-driven, evidence-based approach, and has strengthened local relationships among groups with common research interests. These efforts have helped feed growing interest in additional analyses of the effects of temperature on public health in Ahmedabad and Gujarat more broadly.

### 3.4. Coalition Building and Outreach

During the HAP’s development, and after its official announcement by the Municipal Commissioner and Mayor of the City, the AMC continued to reach out to partner organizations and new constituencies in Ahmedabad. These included members of the media, employers and worker organizations, other municipal and state agencies involved in emergency preparedness and response, and affected community members, to assure that Plan implementation would be robust. Members of the coalition presented information about the Plan at international meetings and regional conferences [19], expanding its reach beyond Ahmedabad municipal and Gujarat state boundaries.

### 3.5. Intervention Development

Here we report findings related to the HAP and the heat early warning system.

#### 3.5.1. Heat Action Plan

The 2013 HAP focuses on three pilot strategies:

1. *Community Outreach to Build Public Awareness* of the risks of heat waves and practices to prevent heat-related deaths and illnesses. Disseminating messages through media outlets and informational materials such as pamphlets and advertisements on heat stress prevention, with tips for health protection during extreme heat events, is critical to building awareness. The complete HAP was also adapted into an Easy Read Version for broader dissemination [31].
2. *Initiating a Simple Early Warning System* to alert residents of forecasted dangerously high temperatures and coordinating an inter-agency response effort when extreme heat hits. Established, formal communication channels among relevant agencies, responders, providers, community groups, and media ahead of forecasted high temperatures is important.
3. *Capacity Building among Health Care Professionals* to recognize and respond to heat-related illnesses. Training should focus on primary medical officers so they can accurately educate, diagnose, and manage patients and staff. Training should also address decision making regarding staffing for increased health care demand, surveillance, and reporting of heat illnesses and deaths. Community health workers should be trained to recognize heat-health dangers, provide health education, and facilitate surveillance in vulnerable communities.

The HAP developed by the Ahmedabad Municipal Corporation and the group [31] brings together relevant resources, best practices from various countries, and strategies to minimize health impacts when extreme heat events hit. The Plan’s heat alerts are triggered by temperature thresholds, with flexibility regarding the source of temperature forecast data. Four different color signals corresponding to different levels of heat-health alerts and temperature thresholds, ranging from “no alert” to “extreme heat alert day” (see Supplemental Material and Table S4), are based on the forecasts discussed in the subsection below. 

**Figure 2:**
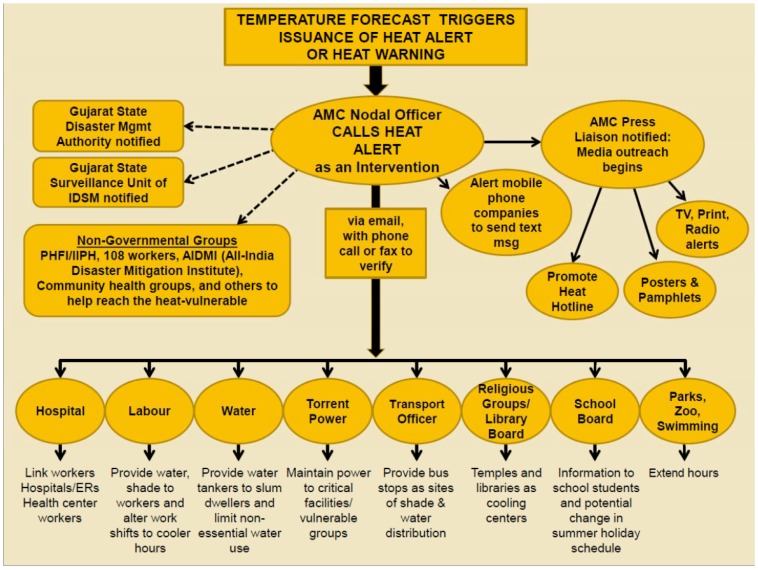
Communications in Ahmedabad’s Heat Action Plan (reprinted from [31] with permission from Natural Resources Defense Council ^©^).

Figure 2 outlines the responsibilities of each agency. As the lead, the Health Department is responsible for overarching response coordination, including monitoring forecasts and sending heat health alerts and disseminating public health messages to local departments and community service providers, as well as working with the Ahmedabad Municipal Corporation press office to increase media around preparedness and warnings. The HAP serves as a master guide to city officials at Ahmedabad Municipal Corporation and the community by outlining strategies for coordinated government agency action on a range of levels, including interventions targeting vulnerable populations and organizational management of response and surveillance activities. 

#### 3.5.2. Extreme Heat Early Warning System

The early warning system was developed to produce probabilistic forecasts of maximum temperature together with probabilities that the temperature will reach specified *critical thresholds* based on group consensus after analysis of mortality and temperature data [12]. The forecasts were generated with a seven-day advance lead time and sent via e-mail to the AMC Nodal Officer as shown in Figure 2. 

### 3.6. Intervention Implementation

As noted, 2013 was a pilot for the intervention implementation to ensure that the forecasting and forecast communication went smoothly and that the HAP could be implemented as planned.

#### 3.6.1. Extreme Heat Early Warning System

**Figure 3:**
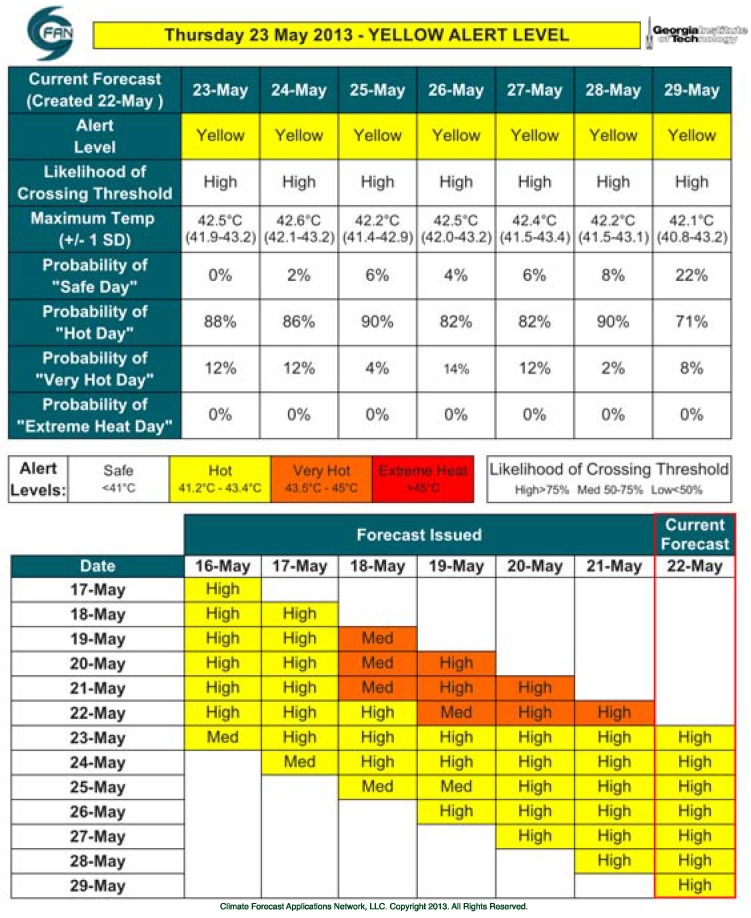
Sample of Maximum Temperature Forecast made on 22 May 2013. The alert level is determined by the likelihood of exceeding specific thresholds, determined by the study group. Note that in this case the forecast shows high probability of “Hot” alert level (temperatures ranging between 41.2 °C and 43.4 °C) for the entire period. Summary of the alert levels issued for the previous 7 days. Note the forecast evolution for the 18–21 May period where the forecast captured the transition to “Very Hot” alert level 3 days in advance. Moreover the forecast captured high likelihood for “Hot” conditions 7 days in advance, throughout the entire period.

Model verification showed significant forecasting skill at all lead times. Seven days’ lead time was identified during planning discussions with AMC as most useful to provide early warning and mount a coordinated public health and inter-agency response. During the first year of the HAP, 7-day predictions provided high accuracy. Preliminary verification results (unpublished data 2014) show the forecast root mean square error (RMSE) going from just under 0.8 °C with a 1-day lead time, to just over 1.2 °C at a 7-day lead time. Moreover, by using an ensemble approach, the current forecast method produces a range of probabilities for various alert levels, and confidence intervals for the average forecast temperature computed based on ensemble spread and historical forecast error. This probabilistic information can be used as guidance for decisions that require lead times longer than 3 days. For example, if the forecast (7 days in advance) shows high probability of an approaching “Extreme Heat Day” and the confidence level is high, planning strategies can be triggered. Temperature forecasts, along with a second panel that provided the outlook summary for the previous 7 days, were shared via daily automatic email alerts. This information was included as the team felt that it could be important for decision makers as it shows forecast consistency and highlights threat levels. An example of the daily forecasts received by the Ahmedabad Health Department from Georgia Tech is shown in Figure 3. 

#### 3.6.2. Communications

When the pilot plan was initiated, the city’s mayor and commissioner convened two separate media events, a media workshop and a press release. At the workshop, study group members highlighted the importance of the media in disseminating information. A series of print advertisements and communication materials were produced in both English and Gujarati.

**Figure 4:**
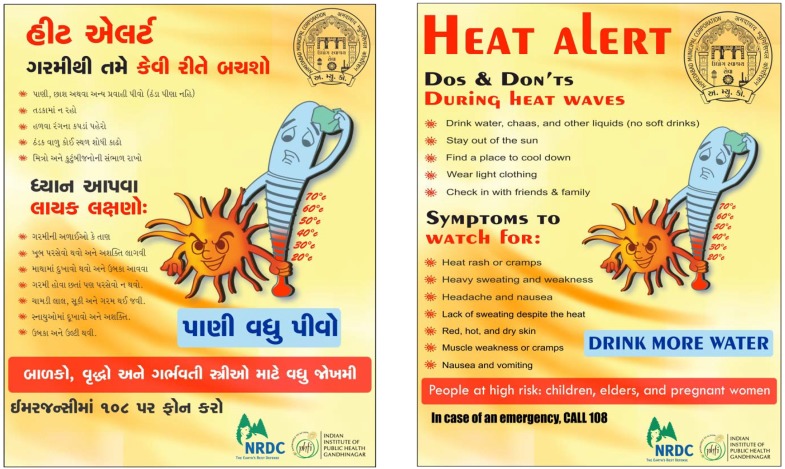
Advertisements Developed for Urban Health Centers (reprinted from [31] with permission from Natural Resources Defense Council ^©^).

The Ahmedabad Municipal Corporation held a series of outreach activities as part of the HAP launch, including billboards around the city with instructions on how to “Save Yourself from Heat”, distributing thousands of pamphlets to schoolchildren and urban health centers with heat-illness prevention tips (see Figure 4) as well as developing a radio campaign in local languages. The city installed electronic temperature displays, one of the first in India, to alert communities of the current temperature and to allow residents to prepare for the heat ahead. 

### 3.7. Project Evaluation

Project evaluation is ongoing. The group has pursued continuous quality improvement efforts throughout the effort, and several lessons have already been learned. Some insights from the study group on what proved to be effective elements of the project are presented in Table 1. 

**Table 1:** Lessons Learned: description of challenges that the project faced, and the team’s response.

Challenge	Description	Response	Looking Forward
Recognition of heat as a disaster and growing health threat	Heat waves were not initially considered disasters requiring high-level preparation.	Heat adaptation plans adopted by other cities offered best practices adaptable to the Indian context.	City leaders have taken ownership and are implementing a comprehensive response to extreme heat.
Interagency communication and coordination	Limited communication between municipal agencies; hospitals and the general public were not alerted to impending heat waves.	Leveraging national, state, and local infrastructure already in place facilitated plan implementation.	Improving formal communication channels and designating an AMC “nodal” (lead) officer to direct heat-related efforts.
International team coordination	Challenges communicating across time zones and in different languages.	Weekly calls with the international team, combined with regular visits to India.	Technologies, such as Skype, and leadership and commitment contributed to the team’s effectiveness.
Data collection	Collecting temperature data and mortality data was challenging.	Developed relationships with meteorological and health agencies to get access to information.	Will streamline the process of instituting heat data collection, making it easier to evaluate the HAP.
Budgetary concerns and political will	Resources are precious and are allocated judiciously; city engagement and leadership were crucial.	Project partners identified and prioritized policies and programs that would have the biggest impact.	Study group working with AMC nodal officer to measure these programs’ effectiveness and hope to add more programs.

Additional evaluation activities are being planned, specifically impact assessment and efficiency characterization. Impact assessment of preparedness activities in the public health community will include focus group discussions and surveys with key personnel involved in anticipating and responding to extreme heat events, to evaluate their understanding of roles and responsibilities, health impacts of extreme heat, appropriate preparedness and response activities, recognition of heat illness syndromes, and barriers to implementation. Key personnel include the local meteorological service, administrators of hospitals and health centers, and administrators of emergency medical services. Results from these focus groups will be compared to findings from focus groups done prior to the project’s implementation. 

Impact assessment in the general population will include: post-intervention surveys of vulnerable populations (e.g., slum households); review of emergency medical service, hospital, and clinic records; and evaluation of post-intervention all-cause mortality records for the city. Self-reported rates of heat illness and prevalence of hot weather coping behaviors in slum dwellers will be compared to baseline rates collected prior to the intervention, from emergency medical service calls, visits to clinics, and hospital admissions for all-causes and heat-specific causes. 

Once impact evaluations have been done it will be possible to evaluate the efficiency and cost effectiveness of the intervention. The Plan was designed to build upon existing capacities, to make the needed budget more manageable. For example, AMC already had billboards available to post public service messages. It was more a matter of developing the political will to make heat vulnerability reduction a municipal priority. Certain assumptions will have to be made regarding the costs of various inputs such as labor and start-up costs for the project, as well as assumptions regarding the costs associated with morbidity and mortality from extreme heat. Few such evaluations of heat early warning systems exist currently, but what has been done suggests that such systems are highly cost effective. Regardless of the efficiency estimates for the program, it is clear that there are potential economies of scale for forecasting and other efforts that could be achieved if similar interventions using the same forecasting inputs are used in other cities in Gujarat and nearby Indian states, and if forecasting were to be expanded to other hazards such as storms and flooding.

## 4. Discussion

In two years, the Ahmedabad Heat and Climate Study Group has accomplished many of its initial goals and objectives. As described and cited in the Results section, the preliminary data collection and research completed in the course of these initial years of this effort has been substantial. While the more academic results provide the means for the international community to learn about the Ahmedabad work, local coalition building, outreach and ongoing coordination among government officials, health practitioners, at-risk communities and public outreach specialists has been critical. As described above, the group has developed and piloted a heat action plan with extensive outreach to the public and media on the health risks of extreme heat; enhanced interagency coordination among municipal government, public health and medical leaders; and implemented a novel early warning system that forecasts extreme heat days with longer lead times that allow a wider variety of public health preparedness activities to be undertaken and coordinated. While much work remains, the effort has had a strong start.

The group attributes its success to several factors, the most important of which is likely the team’s composition. The group’s success was greatly facilitated by the motivation of the Ahmedabad city managers and a network of superb on-the-ground partners in Ahmedabad, led by PHFI/IIPH-G and a team of highly capable student and doctoral candidates. NRDC and other U.S. research partners at Emory University, the Icahn School of Medicine at Mount Sinai, and Georgia Tech also provided useful structure for the team’s efforts. Strong leadership and engagement from the Ahmedabad Municipal Corporation, including the Mayor and the senior health administrators, provided critical credibility for the project, positive local media, and heightened participation across the local municipal agencies. 

Communication has been particularly challenging but also uniquely important. Given that team member locations spanned Ahmedabad, Atlanta, New Delhi, New York, and San Francisco; that telecommunications capacity within the organizations varied; and that team members spoke several different primary languages, communication was often a challenge. Technologies, such as Skype and teleconferencing, proved useful, and in-person visits to Ahmedabad were invaluable. During the course of the project, especially during in-person meetings in Ahmedabad, the team developed a trusted relationship that allowed for frank discussions about public attitudes and perceptions about relevant stakeholders, organizations and working methodologies that are best known within the local community. Ensuring coordinated and continuous communication also addressed the tension that can arise between the need to keep the project moving ahead, and the additional time required to build capacity within partner teams to come up to speed. 

Looking ahead to the next phase of this project, in addition to ongoing evaluation, there are three important priorities: continuous improvement as additional components of the project are brought online, expanding research into the impacts of extreme heat and air pollution on health, and applying lessons learned to other regions in South Asia that may experience similar risks and potentially to management of other environmental, climate-sensitive hazards.

The project appears to have made gains in protecting public health in the face of extreme heat, but it remains to be seen how the HAP will perform during a true extreme heat event. It also remains to be seen whether the resources will be available to implement a wider range of health protection activities, including training children and new mothers on how to keep themselves safe from extreme heat, advancing green buildings, creating additional shaded bus stops, increasing interagency communication, improving hospital protocols for heat-related illness diagnosis and treatment, modifying labor policies, and other strategies. The project’s full potential will be dependent not only on continued successful implementation of the activities underway but also on expansion to include these other measures.

Continued integration of the best available evidence, as well as contribution of project findings to the evidence base, are also key. In the project’s next phase, assessing the burden of heat illness on vulnerable populations in Ahmedabad will apply both retrospective and prospective cohort methods that build on existing community health infrastructure. This will lay the groundwork for (1) an ongoing surveillance-intervention program; (2) an assessment of current adaptive capacity in Ahmedabad’s public health and health care delivery systems to medically manage heat illness and incorporate previously-conducted stakeholder focus group findings; (3) a study of the health effects of air pollution related to heat waves in Ahmedabad*,* because while effects of heat on mortality have been robust to confounding by air pollution [32,33] the possibility exists for confounding of heat-related mortality by air pollution, and future research could investigate this question; (4) modeling of relationships between meteorological variables such as maximum and minimum temperature and relative humidity as exposures associated with heat stress and mortality; and (5) projections of adverse health impacts of extreme heat under various climate change scenarios. 

While our findings center on Ahmedabad, the Plan offers practices that can apply to cities across India, and cities around the world. State officials in Gujarat have discussed scaling up strategies developed in Ahmedabad throughout the state as part of the Gujarat State Action Plan. Key cities such as Jaipur, Bangalore, Chandigarh, and Hyderabad have expressed interest in expanding this project to their cities, and some outreach and exchange of information has already taken place. New challenges loom in determining heat stress thresholds and warning strategies in cities in the coastal margins (e.g., Mumbai, Madras, Kolkata). In such cities, extreme heat exists before the monsoon rains, as in Ahmedabad, though heat hazards related to moderately high temperatures coupled with high humidity, which reduces the body’s ability to dissipate heat, may also pose significant public health challenges. 

Scaling-up activities could include focused discussions with government and civil society leaders in interested cities, development of formal information-exchange opportunities like workshops and briefings, and development of briefing materials that showcase the Ahmedabad experience such as a resources guide, conference presentations, and webinars. Furthermore, the devastating flooding in 2013 from the Indian monsoon underscored the need for preparedness, forecasting and response for other extreme events in the face of climate change, which could mean expanding our set of implementing partners. Gujarat’s mission to implement climate-compatible strategies, reduce urban heat island effects, reorganize and better equip hospitals, enable and safeguard the outdoor workforce, and preserve and increase green cover and efficient town planning may provide collaborative opportunities.

## 5. Conclusions

The Ahmedabad Heat and Climate Study Group project progressed from a novel concept advanced at a meeting in 2009 to a specific set of activities focused on the city of Ahmedabad in 2013. The project was developed based on Gujarat’s interest in climate change generally and the interests and priorities of Ahmedabad’s public health leadership. The collaborative project partnership had the benefit of a high degree of local engagement from municipal leadership, interdisciplinary engagement from scientific partners in India and the U.S., and a toolkit of health research, outreach and advocacy tools that helped capitalize on Ahmedabad’s strong network of health worker-community relationships. In just two years, the group worked with a broad range of partners to pilot the first heat-health action plan and early warning system in India. The project builds an evidence base for publications and sharing information with the local and international community, and may serve as a template for other such projects to help reduce heat vulnerability, especially in lower-resource settings. 

## Abbreviations

AMC: Ahmedabad Municipal Corporation
HAP: Heat Action Plan
IIPH-G: Indian Institute of Public Health-Gandhinagar
IMD: India Meteorological Department
NRDC: Natural Resources Defense Council
PHFI: Public Health Foundation of India

## Supplementary Files

Supplementary File 1 Supplementary Information (PDF, 352 KB)
