# Supplementary material for: Development and Implementation of South Asia’s First Heat-Health Action Plan in Ahmedabad (Gujarat, India)
Source: Int J Environ Res Public Health. 2014 Mar 25;11(4):3473–92. doi: 10.3390/ijerph110403473 (PMC4024996; doi:10.3390/ijerph110403473)
Supplement: Supplementary File 1 — Supplementary Information (PDF, 352 KB) [file ijerph-11-03473-s001.pdf]

Supplementary Information

Development and Implementation of South Asia's First Heat-Health Action Plan in Ahmedabad (Gujarat, India)

Climate of Ahmedabad

Ahmedabad is located in the arid northwest region of India, where warm, dry conditions are conducive to heat waves. While summer is defined as spanning March, April, and May, Ahmedabad's hottest temperatures can run from March through June, with temperatures generally peaking in May and warm days through November. Ahmedabad's average monthly maximum summer temperature from March through June is 38.8 °C (101.8 °F); in winter, November through February, the average monthly high is 28 °C (82 °F). The average monthly minimum temperature is 24 °C (75 °F) in the summertime and 15 °C (59 °F) in the winter. Temperature peaks in Ahmedabad can be extreme, as experienced during the May 2010 heat wave when the temperature spiked to 46.8 °C (116 °F).

City Selection

After identifying extreme heat as the research focus, NRDC-PHFI team examined and developed a matrix of cities willing to engage on scientific research and take leadership on fighting climate change. The city selection process considered whether each municipality had exposure to climate change health risks and heat vulnerability; active health department with resources tracking deaths; partnerships among civil society organizations, public health groups, emergency communication agencies, media and government; interest in infrastructure improvement; appropriate size; on-the-ground PHFI presence, relation and capacity, and ability to develop a replicable program. Our city selection matrix (Table S1), developed during 2010, examined various cities across India for relevant characteristics that would enhance partnerships to develop an effective heat-health adaptation plan.

Table S1. City Selection Matrix Headers.

**Matrix Variability Example**							
City	PHFI Presence or Relationship in City; Capacity	Exposure to climate change health risks; Heat vulnerability	Health Dept. Resources and Administration (track deaths); High environmental awareness	Existence of successful partnerships between environmental, public health, and emergency communication agencies	Interest in infrastructure improvements	Appropriate Scale (size)	Replicable program

Needs Assessment Activities

To provide detail on activities through which needs assessments were conducted, Table S2 provides details on activities, lead organizations and partners, methodological approaches, and project outputs.

Table S2. Project needs assessment activities.

Activity	Lead Organization	Major Partners	Approach	Outputs
Assessment of household factors affecting vulnerability to heat	EUSPH/EUSM	NRDC, IIPH-G/PHFI, MSSM	Randomized cluster sampling of 300 slum households	[1]
Assessment of the 2010 heat wave's impact on all-cause mortality in Ahmedabad	IIPH-G/PHFI	EUSPH/EUSM, MSSM, NRDC	Retrospective evaluation of all-cause mortality associated with 2010 heat wave compared with baseline	[2]
Assessment of the 2010 heat wave's impact on cause-specific morbidity in Ahmedabad	IIPH-G/PHFI	EUSPH/EUSM, MSSM, NRDC	Retrospective evaluation of ambulance calls and selected hospital visits during 2010 heat wave	[3]
Assessment of the 2010 heat wave's impact on call-cause mortality in a neonatal ward	MSSM	NRDC, IIPH-G/PHFI	Retrospective analysis of an epidemic of neonatal mortality during the heat wave	[4]
Assessment of the health sector's capacity to respond to heat emergencies	NRDC	EUSPH/EUSM, MSSM, IIPH-G/PHFI	Focus group discussion with health care providers regarding diagnosis and management of heat illness	[5]
Assessment of occupational heat exposures in construction sites	IIPH-G/PHFI	EUSPH/EUSM, MSSM, NRDC	Focus groups and on-site measurements of heat exposure at outdoor construction sites	[6,7]
Assessment of thresholds for heat early warnings	IIPH-G/PHFI	EUSPH/EUSM, MSSM, NRDC	Retrospective analysis of sensitivity and specificity of various temperature thresholds for various indicators of heat-health impacts	[8]

Heat Action Plan Strategies

The 2013 Ahmedabad Heat Action Plan is the first comprehensive early warning system and preparedness plan for extreme heat events in South Asia. The Plan creates immediate and longer-term actions to increase preparedness, information-sharing, and response coordination to reduce the health impacts of extreme heat on vulnerable populations. The initial activities under the Heat Action Plan focus on three key strategies:

- Building Public Awareness and Community Outreach on the risks of heat waves and practices to prevent heat-related deaths and illnesses.
- Initiating a Simple Early Warning System to alert residents of predicted high temperatures, and coordinating an inter-agency response effort when extreme heat hits.
- Capacity Building among Health Care Professionals to recognize and respond to heat-related illnesses, particularly during extreme heat events.

Some of the major project milestones, key study team members, and funding organizations over the course of the project are depicted in Table S3.

Table S3. Major milestones, primary actors and funders in the course of the project.

Date	2008	2010	2011	2012	2012	2012	2012	2013
Milestone	Goa Climate and Health Meeting	Ahmedabad Heat Meeting	AMC Kick-off workshop	AMC Training	Heat Vulnerability Study	Heat Early Warning System	AMC Heat Action Plan Development	Dry-run Heat Action Plan
Location	Goa	Gandhinagar	Ahmedabad	Ahmedabad	Ahmedabad	Atlanta	Ahmedabad	Ahmedabad
Primary Actors	CDC; University of Michigan; US-Indo Collaborative; IIPH-G/PHFI; NRDC	Ahmedabad Municipal Corporation (AMC); NRDC; IIPH-G			IIPH-G; Emory; NRDC; Icahn School of Medicine/Mt.Sinai	Georgia Institute of Technology and Climate Forecast Applications Network		
Funder(s)	CDC, US-Indo	US-Indo, NRDC	CDKN, NRDC	CDKN, NRDC	Emory; CDKN	CDKN	CDKN; NRDC	CDKN; NRDC

An alternative way to characterize implementation is by project objectives as outlined in Figure S1 below, which details the project's objectives within its overall multi-year timeline structure.

Figure S1. Project objectives.

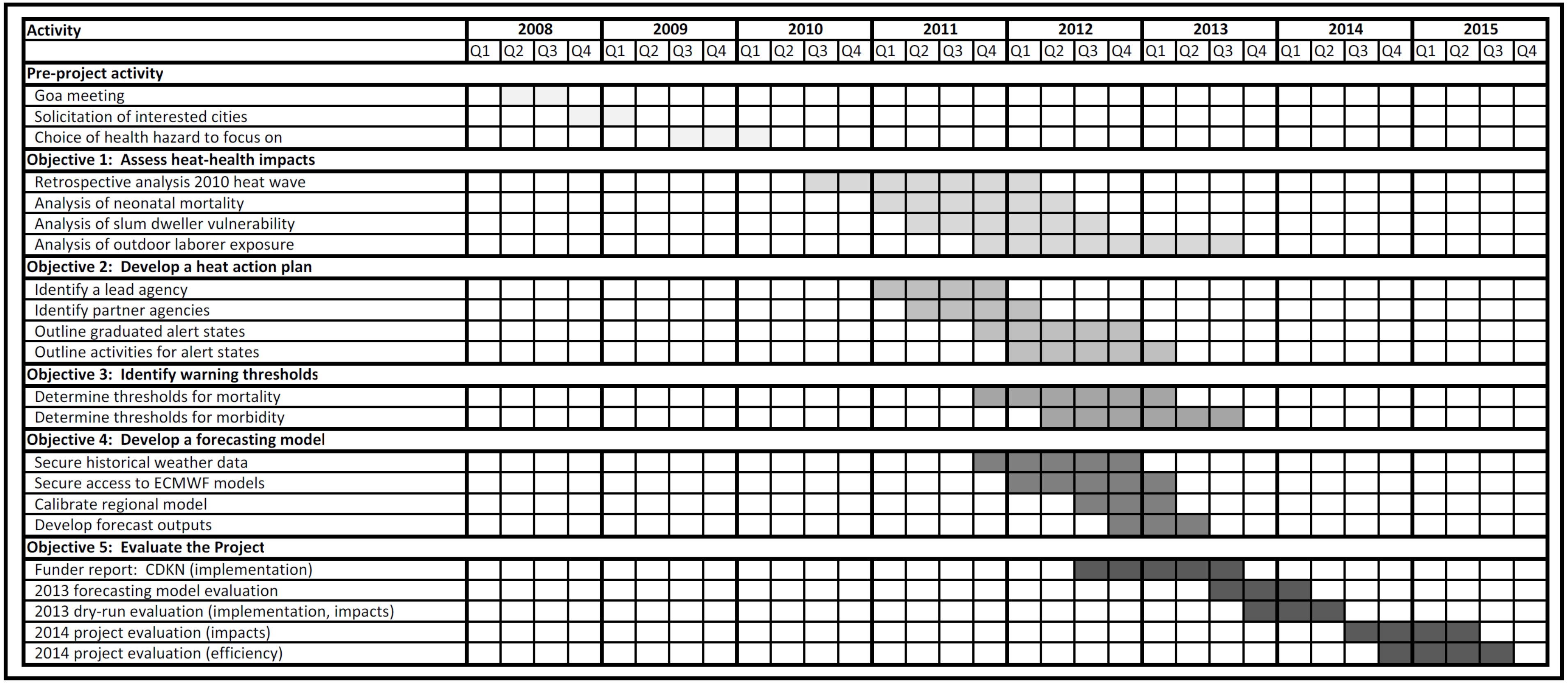

Extreme Heat Early Warning System

The Early Warning System consists of heat alerts that are triggered by specific temperature thresholds, correlated with four different colors applied in public messaging (see Table S4):

Table S4. Heat-health Early Warning system, color coding and temperature thresholds.

Color Code	Public Message	Daily Maximum Temperature
WHITE	No Alert	<41 °C
YELLOW ALERT	Hot Day Advisory	41 °C–43.4 °C
ORANGE ALERT	Heat Alert Day	43.5 °C–45 °C
RED ALERT	Extreme Heat Alert Day	>45 °C

The early warning system currently involves four levels: "No Alert"; "Hot Day Advisory"; "Heat Alert Day"; and "Extreme Heat Alert Day" with color signals.

Note: More Supplementary Materials about these and other aspects of the program, including the four Policy Briefs, can be found at the Natural Resources Defense Council's India Initiative website [9].

Reference

1. Tran, K.; Azhar, G.; Nair, R.; Knowlton, K.; Jaiswal, A.; Sheffield, P.; Mavalankar, D.; Hess, J. A cross-sectional, randomized cluster sample survey of household vulnerability to extreme heat among slum dwellers in Ahmedabad, India. *Int. J. Environ. Res. Public Health* **2013**, *10*, 2515–2543.
2. Azhar, G.; Mavalankar, D.; Nori-Sarma, A.; Rajiva, A.; Dutta, P.; Jaiswal, A.; Sheffield, P.E.; Knowlton, K.; Hess, J. Heat-related mortality in India: Excess all-cause mortality associated with the 2010 Ahmedabad heat wave. *PLoS One* **2014**, *93*, doi:10.1371/journal.pone.0091831.
3. Azhar, G.; Mavalankar, D.; Kulkarni, S.P.; Rajiva, A.; Dutta, P.; Knowlton, K.; Jaiswal, A.; Deol, B.; Sheffield, P.; Hess, J.; *et al*. Assessment of the 2010 heat wave's impact on cause-specific morbidity in Ahmedabad **2014**, to be submitted.
4. Kakkad, K.; Barzaga, M.; Wallenstein, S.; Shah Azhar, G.; Sheffield, P.E. Neonates in Ahmedabad, India during the 2010 heat wave: A climate change adaptation study. *J. Env. Pub. Health* **2014**, *2014*, doi:10.1155/2014/946875.
5. Azhar, G.S.; Casey-Lefkowitz, S.; Connolly, M.; Deol, B.; Dholakia, H.; Gill, G.; Hess, J.; Jaiswal, A.; Khosla, R.; Knowlton, K.; Mavalankar, D.; Rajiva, A.; Sarma, A.; Sheffield, P.; Thube, N.; van Tran, K. Rising Temperatures, Deadly Threat: Recommendations for Health Professionals in Ahmedabad. Available online: http://www.nrdc.org/international/india/extreme-heat-preparedness/files/india-heat-health-professionals-IB.pdf; (accessed on 7 March 2014).
6. Azhar, G.S.; Casey-Lefkowitz, S.; Connolly, M.; Deol, B.; Dholakia, H.; Gill, G.; Hess, J.; Jaiswal, A.; Khosla, R.; Knowlton, K.; Mavalankar, D.; Rajiva, A.; Sarma, A.; Sheffield, P.; Thube, N.; van Tran, K. Rising Temperatures, Deadly Threat: Recommendations to Prepare Outdoor Workers in Ahmedabad. Available online: http://www.nrdc.org/international/india/extreme-heat-preparedness/files/india-heat-outdoor-workers-IB.pdf; (accessed on 7 March 2014).

7. Rajiva, A.; Venugopal, V.; Dutta, P.; Azhar, G.; Knowlton, K.; Sheffield, P.E.; Hess, J.; Mavalankar, D. Heat stress and its perceptions among construction workers in Ahmedabad, India. *J. Environ. Health Res.* **2014**, submitted.
8. Rajiva, A.; Azhar, G.; Mavalankar, D.; Kulkarni, S.P.; Knowlton, K.; Jaiswal, A.; Webster, P.J.; Toma, V.E.; Sheffield, P.; Hess, J.; *et al*. Assessment of thresholds for heat early warnings. **2014**, to be submitted.
9. Natural Resources Defense Council (NRDC). India Initiative Website. http://www.nrdc.org/international/india (accessed on 18 March 2014).

© 2014 by the authors; licensee MDPI, Basel, Switzerland. This article is an open access article distributed under the terms and conditions of the Creative Commons Attribution license (http://creativecommons.org/licenses/by/3.0/).
